# Correlation of anogenital distance from childhood to age 9 years—a prospective population-based birth cohort—the Odense Child Cohort

**DOI:** 10.1093/hropen/hoae050

**Published:** 2024-09-09

**Authors:** Sarah Munk Andreasen, Lise Gehrt, Casper P Hagen, Anders Juul, Gylli Mola, Margit Bistrup Fischer, Marianne Skovsager Andersen, David Møbjerg Kristensen, Tina Kold Jensen

**Affiliations:** Department of Clinical Pharmacology, Pharmacy and Environmental Medicine, Institute of Public Health, University of Southern Denmark, Odense, Denmark; Hans Christian Andersen Research, Hans Christian Andersen Children’s Hospital, Odense University Hospital, Odense, Denmark; Department of Clinical Pharmacology, Pharmacy and Environmental Medicine, Institute of Public Health, University of Southern Denmark, Odense, Denmark; Hans Christian Andersen Research, Hans Christian Andersen Children’s Hospital, Odense University Hospital, Odense, Denmark; Department of Growth and Reproduction, Copenhagen University Hospital—Rigshospitalet, Copenhagen, Denmark; International Center for Research and Research Training in Endocrine Disruption of Male Reproduction and Child Health (EDMaRC), Copenhagen, Denmark; Department of Growth and Reproduction, Copenhagen University Hospital—Rigshospitalet, Copenhagen, Denmark; International Center for Research and Research Training in Endocrine Disruption of Male Reproduction and Child Health (EDMaRC), Copenhagen, Denmark; Department of Clinical Medicine, University of Copenhagen, Copenhagen, Denmark; Department of Growth and Reproduction, Copenhagen University Hospital—Rigshospitalet, Copenhagen, Denmark; International Center for Research and Research Training in Endocrine Disruption of Male Reproduction and Child Health (EDMaRC), Copenhagen, Denmark; Department of Growth and Reproduction, Copenhagen University Hospital—Rigshospitalet, Copenhagen, Denmark; International Center for Research and Research Training in Endocrine Disruption of Male Reproduction and Child Health (EDMaRC), Copenhagen, Denmark; Department of Endocrinology, Odense University Hospital, Odense, Denmark; Department of Clinical Research, Institute of Clinical Research, Faculty of Health Sciences, University of Southern Denmark, Odense, Denmark; Department of Growth and Reproduction, Copenhagen University Hospital—Rigshospitalet, Copenhagen, Denmark; Department of Science and Environment, Roskilde University, Roskilde, Denmark; Department of Clinical Pharmacology, Pharmacy and Environmental Medicine, Institute of Public Health, University of Southern Denmark, Odense, Denmark; Hans Christian Andersen Research, Hans Christian Andersen Children’s Hospital, Odense University Hospital, Odense, Denmark; Odense Patient Data Explorative Network, Odense University Hospital, Odense, Denmark

**Keywords:** anogenital distance, cohort study, childhood, reproductive health, endocrine disruption

## Abstract

**STUDY QUESTION:**

Does anogenital distance (AGD) – distance from the anus to the genitals – correlate from infancy (3 months) to the age of 9 years in boys and girls?

**SUMMARY ANSWER:**

In boys, AGD correlated from infancy to 9 years of age, whereas in girls, correlations were weaker, especially between infancy and later childhood.

**WHAT IS KNOWN ALREADY:**

AGD is considered a marker for prenatal androgen action. In males, reduced AGD is associated with testicular cancer, infertility, and lower sperm count. In females, AGD is associated with endometriosis and polycystic ovary syndrome.

**STUDY DESIGN, SIZE, DURATION:**

In the Odense Child Cohort, a prospective population-based birth cohort, pregnant women were enrolled in early pregnancy. AGD and BMI were measured repeatedly in children at ages 3 and 18 months, as well as at 3, 5, 7, and 9 years.

**PARTICIPANTS/MATERIALS, SETTING, METHODS:**

AGD was measured from the anus to the scrotum (AGDas) and to the penis (AGDap) in 1022 boys, and to the posterior fourchette and the clitoris in 887 girls repeatedly between the age of 3 months to 9 years. In total, 7706 assessments were made. AGD was adjusted for body weight, and *SD scores* (the difference between individual AGD and the mean of AGD in the population divided by SD of AGD) were calculated for each child. Pearson correlation coefficient (*r*) of each measurement was performed to investigate whether individual AGD was stable during childhood. Short predictive values at 3 months (20th percentile) to 9 years were investigated using the AUC produced by the receiver operating characteristic curve.

**MAIN RESULTS AND THE ROLE OF CHANCE:**

In boys, AGD/body size-index *SD score* correlated significantly between infancy and 9 years, strongest for AGDas (*r* = 0.540 *P* > 0.001). In girls, weaker significant correlation coefficients were found between AGD at infancy and 9 years; higher correlation coefficients were found between AGD from 3 to 9 years (*P* > 0.001). Short AGDas in infancy predicted short AGDas in boys aged 9 years (AUC: 0.767, sensitivity 0.71, specificity 0.71). The predictive values of short infant AGDap, penile width (in boys), and AGD (in girls) concerning short outcomes at 9 years were low.

**LIMITATIONS, REASONS FOR CAUTION:**

The AGD measurements are less precisely measurable in girls compared to boys, especially in infancy, resulting in less reproducible measurements. Additionally, because AGD is shorter in girls, the same absolute measurement error is relatively more significant, potentially contributing to greater variability and lower reproducibility in girls. This may contribute to the weaker correlations in girls compared to boys.

**WIDER IMPLICATIONS OF THE FINDINGS:**

In boys, AGDas, relative to body size, correlated from infancy to 9 years, suggesting that AGD in infancy can be considered a non-invasive marker of later reproductive health. Further follow-up studies are needed to evaluate long-term individual tracking of AGD as well as assessment of childhood AGD as early marker of adult reproductive health.

**STUDY FUNDING/COMPETING INTEREST(S):**

This study was supported by Odense University Hospital, Denmark, the Region of Southern Denmark, the Municipality of Odense, Denmark, the University of Southern Denmark, Odense Patient data Exploratory Network (OPEN), Denmark, the Danish Research Council (4004-00352B_FSS), Novo Nordisk Foundation, Denmark (grant no. NNF19OC0058266 and NNF17OC0029404), Sygeforsikring Danmark (journalnr. 2021-0173), the Collaborative Foundation between Odense University Hospital and Rigshospitalet, and Helsefonden. There is no conflict of interest of any author that could be perceived as prejudicing the impartiality of the research reported.

**TRIAL REGISTRATION NUMBER:**

N/A.

WHAT DOES THIS MEAN FOR PATIENTS? The anogenital distance (AGD) – the distance from the anus to the genitalia – is affected by exposure to chemicals that can disrupt hormone function during early development in weeks 8 to 14. A shorter AGD has been linked to reproductive disorders, e.g. lower semen count and testosterone levels and testicular cancer in men and endometriosis and polycystic ovary syndrome in women.

It is unknown if AGD remains consistent throughout childhood. If so, it may serve as a potential early non-invasive marker of reproductive health later in life. Therefore, we studied variations in AGD among children at ages 3 and 18 months and 3, 5, 7, and 9 years.

In boys, AGD correlated through childhood, suggesting that AGD in infancy could serve as an early marker of future reproductive health. In girls, correlation of AGD was strongest from 3 years to 9 years. AGD is easily obtained and may be included in studies of children to monitor reproductive health and population trends. However, individual AGD measurements need further investigations before recommending diagnostic tests for children with short AGD.

## Introduction

Anogenital distance (AGD) is an indicator of androgen activity *in utero* during a critical period of gonadal and genital development known as the ‘masculinization programming window’ ranging from gestational weeks 8–14 (Dean and Sharpe, 2013; Welsh *et al.*, 2014; Thankamony *et al.*, 2016). As a consequence of the androgen-driven dimorphic sex differentiation, male AGD is approximately twice as long as female AGD (Greenham and Greenham, 1977; Welsh *et al.*, 2008). In rodents, foetal exposure to endocrine-disrupting chemicals with anti-androgenic properties reduces AGD in males, and the same associations are observed in human studies (Swan *et al.*, 2005; Bustamante-Montes *et al.*, 2013; Curi *et al.*, 2023). A U.S. Environmental Protection Agency guideline has identified AGD as an endpoint for reproductive toxicity in human studies (Diamanti-Kandarakis *et al.*, 2009). In adult men, a shortened AGD is associated with reproductive disorders, e.g. cryptorchidism, hypospadias, lower sperm count, serum testosterone levels, infertility, and testicular cancer (Skakkebaek *et al.*, 2001). However, these associations have been reported in cross-sectional studies, and it remains to be elucidated whether AGD is a stable marker of male reproductive health from infancy through childhood and puberty to adulthood.

In adult males, individual AGD remains stable over time (Eisenberg *et al.*, 2013). Age-related changes in AGD during the first year of life have been assessed in longitudinal studies (Thankamony *et al.*, 2009; Papadopoulou *et al.*, 2013; Priskorn *et al.*, 2018; Fischer *et al.*, 2020). In both sexes, AGD increased rapidly from birth to 3 months and then plateaued after 1 year of age (Thankamony *et al.*, 2009; Papadopoulou *et al.*, 2013). However, these studies did not adjust AGD for body size.

The impact of AGD on reproductive health is less investigated in females (Schwartz *et al.*, 2019). However, in female rodents, prenatal exposure to anti-androgenic chemicals, e.g. certain phthalates and pharmaceuticals, resulted in shorter and more feminine AGD (Wolf *et al.*, 2002; Dean *et al.*, 2012; Holm *et al.*, 2016). Longer AGD in adult women has been associated with higher serum testosterone levels, increased number of ovarian antral follicles (Mendiola *et al.*, 2012; Mira‐Escolano *et al.*, 2014), and risk of polycystic ovary syndrome (PCOS) (Barrett *et al.*, 2018; Peters *et al.*, 2020; Prieto-Sánchez *et al.*, 2022). Shorter AGD has been linked to endometriosis (Kim *et al.*, 2015; Mendiola *et al.*, 2016; Sánchez‐Ferrer *et al.*, 2019; Peters *et al.*, 2020).

In a previous study from the Odense Child Cohort (OCC), we found that AGD at 3 and 18 months correlated and that variation between and within-observers was low (Priskorn *et al.*, 2018). We have measured AGD repeatedly at ages 3 and 18 months and at 3, 5, 7, and 9 years. In the present study, we expand the longitudinal evaluation to include repeated measures of AGD from infancy (3 months) to age 9 years.

## Materials and methods

The study population is part of the OCC, an ongoing population-based cohort, described in detail elsewhere (Kyhl *et al.*, 2015). In brief, pregnant women residing in the Municipality of Odense, Denmark, between January 2010 and December 2012, were invited to participate in the cohort during the routine ultrasound examination conducted between gestational weeks 10 and 16 at Odense University Hospital. A total of 2448 singletons were enrolled in the cohort. Information on gestational age, child sex, birth weight, and length were derived from birth records. The children were invited to participate in the first examination at 3 months after the expected date of birth and at 18 months and 3, 5, 7, and 9 years. At the clinical examinations, height, weight, and AGDs were measured (Kyhl *et al.*, 2015). Children with AGD measurements from more than one examination were included in the present study.

### Anogenital distance

AGD measurements were made according to the standardized methods established for ‘The Infant Development and the Environment Study’ (TIDES) (Sathyanarayana *et al.*, 2015). The children were placed on a flat surface and positioned with legs held back in a frog leg posture at a 45°–60° angle from the torso at the hips (Sathyanarayana *et al.*, 2015). Measurements were performed using a Vernier caliper with numbers facing away from the examiner. The measurements were repeated three times without closing the caliper in between and an arithmetic mean was calculated. In boys, AGD was measured from the centre of the anus to the posterior base of the scrotum (AGDas) and to the cephalad insertion of the penis (AGDap). Penile width was also measured at the base of the penis. In girls, AGD was measured from the centre of the anus to the posterior fourchette (AGDaf) and to the top of the clitoris (AGDac). A total of eight technicians were involved in handling AGD and penile width measurements. However, 80.8% of the examinations were performed by three technicians (33.1%, 24.4%, and 23.3%). The technicians were specially trained and supervised to achieve the highest possible accuracy. The accuracy of the first 46 AGD measurements in girls was low due to the inconsistency of using specific anatomical landmarks. The technicians received further training to make sure that measurements were performed adequately; however, the first 46 AGD measurements in girls were excluded from the present analyses.

### Statistics

Previous studies have identified body weight as a correlate of AGD (Thankamony *et al.*, 2009; Eisenberg *et al.*, 2013; Papadopoulou *et al.*, 2013). Thus, we calculated an AGD/body size-index by dividing AGD measurement with weight of the child (kg). We then calculated a *SD score* for each of the six examinations as the difference between the individual AGD/body size-index of each child and the mean of AGD/body size-index of the children with the two AGD measurements we will compare, divided by SD of the AGD/body size-index. The calculations were performed for each of the four AGD measurements. The same calculations were conducted for penile width. See the calculation below:

$$\mathrm{AGD}/\mathrm{body}\;\mathrm{size}-\mathrm{index}\;SD\;\mathrm{score}=\frac{\mathrm{AGD}/\mathrm{body}\;\mathrm{size}-i{\mathrm{ndex}}_{t}-\mathrm{mean}\;\mathrm{of}\;\mathrm{AGD}/\mathrm{body}\;\mathrm{size}-i{\mathrm{ndex}}_{t,i}\;}{SD\;\mathrm{AGD}/\mathrm{body}\;\mathrm{size}-i{\mathrm{ndex}}_{t,i}}$$

AGD/body size-index_*t*_: AGD at examination, e.g. at 3 months,

AGD/body size-index_*i*_: Children with AGD measures in the comparison category, e.g. at 18 months, and

AGD/body size-index_*t*__,__*i*_: Children included in correlation plot with measures at both examinations, e.g. at 3 months and 18 months.

First, the different AGD/body size-index *SD scores* at different examinations were plotted against each other and correlation coefficients between examinations were evaluated by Pearson correlation coefficient. Second, we divided the children into tertiles for each AGD at every examination based on individual mean of AGD/body size-index *SD scores* from all longitudinal measurements at each examination. We then investigated the trajectory of the individual AGD measurement from each examination in relation to the child’s average tertile. Third, we conducted a receiver operating characteristic curve to test the predictive value of shorter AGD (20th percentile) in infancy and short AGD in children aged 9 years. The analyses provide AUC, sensitivity, and specificity for predicting short AGD at 9 years.

Data were analysed using Stata 17 (StataCorp. 2021. Stata Statistical Software: Release 17. College Station, TX, USA: StataCorp LLC).

### Ethical approval

The study was performed in accordance with the Helsinki Declaration II and approved by the Regional Ethical Review Committee (Project ID S-20090130) and the Danish Data Protection Agency (j.no. 18/15692) (Kyhl *et al.*, 2015). All participants received written and oral information and provided their written consent for participation (Kyhl *et al.*, 2015).

## Results

From a total of 2448 children included in the OCC, measurements of AGD in boys were obtained from 4157 examinations in 1022 boys aged 3 months to 9 years (median: 4 (range, 2–6) measurements per boy) and AGD measurements in girls were obtained from 3549 examinations in 887 girls aged 3 months to 9 years (median 4 (range, 2–6) measurements per girl).

**Figure 1.**
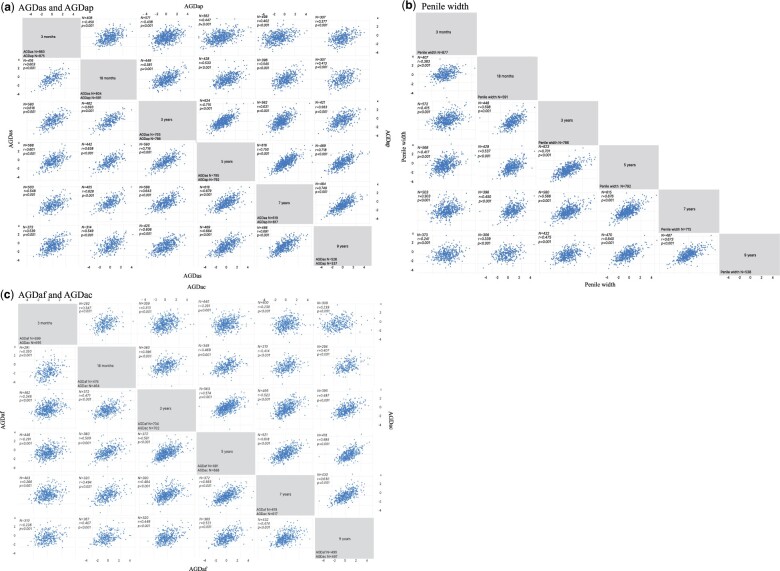
**Correlations between different ages (3 and 18 months, 3, 5, 7, and 9 years) in AGD/body size-index *SD score* and penile width/body size-index *SD score* in boys (a, b) and girls (c).** (a) AGDas (left and bottom) and AGDap (right and top), (b) Penile width, and (c) AGDaf (left, bottom) and AGDac (right and top). *r*, correlation coefficients; *P*, *P*-value; AGD/body size-index, AGD in relation to child weight; *SD score*, the difference between the individual AGD/body size-index of each child and the mean of AGD/body size-index of the population divided by SD of the AGD/body size-index; AGD, anogenital distance; AGDap, anogenital distance from anus to penis; AGDas, anogenital distance from anus to scrotum; AGDac, anogenital distance from anus to clitoris; AGDaf, anogenital distance from anus to posterior fourchette; Penile width.

AGD/body size-index *SD score* for each child correlated between all the examinations in both boys and girls (*P *<* *0.001) (Fig. 1a and c). High correlation coefficients were consistently observed for AGD in boys across different age groups. Measurements of AGDas in boys aged 3 and 18 months, 3, 5, and 7 years correlated strongly with AGDas in boys aged 9 years (*r* = 0.539, *r* = 0.549, *r* = 0.606, *r* = 0.664, *r* = 0.691, respectively) (Fig. 1a). High correlation coefficients were also found for AGDap; 3, 5, and 7 years correlated with 9 years with *r* = 0.563, *r* = 0.718, *r* = 0.749, respectively. Less robust coefficients were observed for AGDap when comparing later measurements with AGDap at 3 and 18 months of age; the weakest correlation coefficient was between 3 months and 9 years (*r* = 0.377).

Penile width measured at different ages correlated between all examinations (all *P* < 0.001) (Fig. 1b). Correlation coefficients varied from the highest value of 0.701 observed between boys aged 3 and 5 years to the lowest of 0.241 observed between boys aged 3 months and 9 years of age.

Generally, lower correlation coefficients (all *P* < 0.001) were found for AGD measurements in girls than in boys (Fig. 1c). In girls aged 3 months, both AGDaf and AGDac showed low correlation coefficients with measurements at other ages: the highest value of 0.347 observed between AGDac in girls aged 3 months and 18 months to the lowest value of 0.230 between AGDac in girls aged at 3 months to 7 years. Higher correlation coefficients were found for AGDaf between 3 and 5 years, and between 5 and 7 years (*r* = 0.581, *r* = 0.565). AGDac in girls aged 5 and 7 years correlated with higher coefficients in girls aged 9 years (*r* = 0.585, *r* = 0.630).

**Figure 2.**
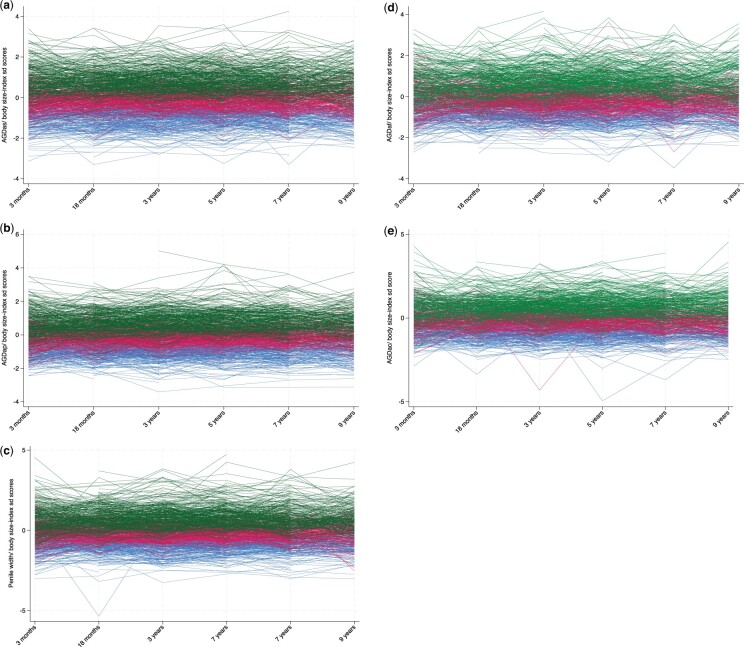
**Trajectories of AGD/body size-index *SD score* and penile width/body size-index *SD score* in boys and girls (from 3 months to 9 years).** Individual AGD/body size-index *SD scores* and penile width/body size-index *SD scores* according to their mean AGD/body size-index *SD score* and penile width/body size-index *SD score* divided in tertiles from children aged 3 months to 9 years in males (**a, b, c**) and females (**d, e**) (blue: shortest, red: middle and green: longest). AGD/body size-index, AGD in relation to child weight; *SD score*, the difference between the individual AGD/body size-index of each child and the mean of AGD/body size-index of the population divided by SD of the AGD/body size-index; AGD, anogenital distance; AGDap, anogenital distance from anus to penis; AGDas, anogenital distance from anus to scrotum; AGDac, anogenital distance from anus to clitoris; AGDaf, anogenital distance from anus to posterior fourchette; Penile width.

The trajectory of measurements relative to tertile of mean AGD/body size-index *SD score* and penile width/body size-index *SD scores* from 3 months to 9 years is shown in Fig. 2. For all measures, the relative AGD and penile width/body size-index *SD scores* were maintained; e.g. if AGD or penile width is short, they will remain so through childhood.

**Figure 3.**
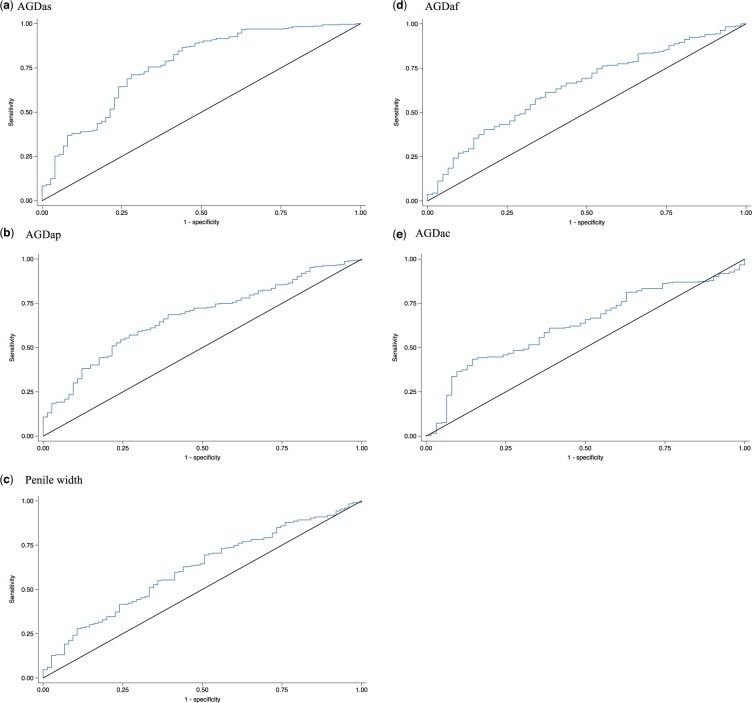
**Receiver operating characteristic (ROC) curves of short AGD and penile width (shortest 20% of mean AGD/body size-index SD score) aged 3 months as predictors of short AGD in children aged 9 years.** (**a**) AGDas: AUC 0.767, sensitivity 0.71, specificity 0.71. (**b**) AGDap: AUC 0.674, sensitivity 0.64, specificity 0.64. (**c**) penile width: AUC 0.615, sensitivity 0.59, specificity 0.59. (**d**) AGDaf: AUC 0.647, sensitivity 0.61, specificity 0.60. (**e**) AGDac: AUC 0.631, sensitivity 0.61, specificity 0.61. AUC, area under the curve; AGD/body size-index, AGD in relation to child weight; *SD score*, the difference between the individual AGD/body size-index of each child and the mean of AGD/body size-index of the population divided by SD of the AGD/body size-index; AGD, anogenital distance; AGDap, anogenital distance from anus to penis; AGDas, anogenital distance from anus to scrotum; AGDac, anogenital distance from anus to clitoris; AGDaf, anogenital distance from anus to posterior fourchette; Penile width.

Short AGD at 3 months (20th percentile) predicted short AGD at 9 years in boys (AGDas: AUC 0.767, sensitivity 0.71, specificity 0.71; AGDap: AUC 0.674, sensitivity 0.64, specificity 0.64) (Fig. 3a and b). Thus, there was a 76.7% chance that short AGDas at 3 months predicted short AGDas at 9 years; 71% of boys with short AGDas at 3 months had short AGDas at 9 years; and 71% of boys not having short AGDas at 3 months still did not have short AGDas at 9 years. Slightly lower predictive values were observed for AGDap. Short penile width at 3 months predicted short penile width at 9 years (AUC 0.615, sensitivity 0.59, specificity 0.59) (Fig. 3c).

In girls, there was a 64.5% and 63.1% chance that short AGDaf and AGDac at 3 months predicted short AGD at 9 years (AGDaf: AUC: 0.645, sensitivity 0.61, specificity 0.60; AGDac: AUC: 0.631, sensitivity 0.61, specificity 0.61) (Fig. 3d and e).

## Discussion

Individual AGD/body size-index *SD scores* remained stable from infancy to 9 years among healthy children from the OCC. AGD was significantly correlated between all timepoints in both boys and girls. Short AGD in infancy predicted short AGD in boys aged 9 years, strongest for AGDas. Penile width/body size-index *SD scores* also remained stable through childhood. While penile width was significantly correlated across all timepoints, similar to the AGD, the correlation coefficients were lower. While AGD/body size *SD scores* in girls were highly correlated from age 3 to 9 years, weaker correlations were observed from infancy to older ages in childhood.

To our knowledge, no previous studies have measured AGD and penile width repeatedly through childhood. A previous study from OCC investigated correlations between AGD and penile width measured at ages 3 and 18 months and reported high correlation coefficients in AGDas and weaker correlations in the other AGD and penile width measurements compared with this study (Priskorn *et al.*, 2018). However, an AGD and penile width/body size-index *SD score* was not calculated, which may explain the weaker correlations. A British cohort study suggests that AGD and penile width measured at birth are not as reliable as AGD and penile width measured later in infancy (Thankamony *et al.*, 2009). The correlation coefficients between birth and up to 2 years were consistently lower compared with our study. In line with the British findings, a cohort study from Greece reported only low correlation coefficients between AGD at birth and at a home visit during the first year of life in both boys and girls (Papadopoulou *et al.*, 2013). Additionally, the study observed weak correlations for penile width. None of the existing studies adjusted for body weight, which may have contributed to the weaker correlations as body weight is identified as a determinant of AGD (Thankamony *et al.*, 2009; Eisenberg *et al.*, 2013; Papadopoulou *et al.*, 2013). The British and Greek cohort studies may suggest that AGD and penile width measured at birth are not as reliable as AGD and penile width measured later in infancy. Unfortunately, OCC does not have AGD measurements from birth, preventing us from evaluating the predictive value of AGD at birth. The ‘mini-puberty’ occurs from birth to 3 months of age and is characterized by a temporary increase in testosterone and other reproductive hormones (Kuiri-Hänninen *et al.*, 2014). In boys, this period is associated with penile and testicular growth (Boas *et al.*, 2006; Pasterski *et al.*, 2015), while effects on AGD have not been studied. We assessed AGD at the end or shortly after ‘mini-puberty’. The more developed genitals at 3 months may explain our higher correlation coefficients compared to the British and Greek cohort studies (Thankamony *et al.*, 2009; Papadopoulou *et al.*, 2013).

AGD is stable throughout life in both male and female rats (Hotchkiss *et al.*, 2007) and is an established lifelong indicator of prenatal androgen action (Welsh *et al.*, 2008; van den Driesche *et al.*, 2011). In rats, short AGD is considered a sensitive marker of anti-androgen exposure *in utero*, particularly during the critical ‘masculinization programming window’, which is identified to be embryonic day 15.5–18.5 (Welsh *et al.*, 2008). Prenatal exposure to anti-androgenic phthalates induces a syndrome of genital dysmorphology in male rats, which has been termed the ‘phthalate syndrome’ (Foster, 2006). This syndrome includes incomplete testicular descent, small penile size, reduced testis weight, reduced semen quality, and shortened AGD (Foster *et al.*, 2001). Though findings in female rats have been less convincing, rodent models have suggested that foetal exposure to phthalates may disrupt the establishment of the primordial follicle pool essential for the reproductive lifespan of the female offspring (Zhang *et al.*, 2013; Hannon *et al.*, 2014). Additionally, some rodent studies found a potential masculinizing effect of androgens as evidenced by an increase in the AGD in female offspring (Wolf *et al.*, 2002; Dean *et al.*, 2012; Holm *et al.*, 2016). However, controversies over these findings exist (Parks *et al.*, 2000). One study of rats assessed AGD repeatedly at 4, 6, 8, and 10 weeks, with a significant association found only at 10 weeks (Holm *et al.*, 2016). This suggests that AGD in female rodents can be more accurately determined as the size of the animals increases (Holm *et al.*, 2016), which is in agreement with our findings where robust correlations were found from age 3 years. This may be explained by the fact that AGD measurements become easier to measure as the distance increases.

Studies suggest that the ‘phthalate syndrome’ observed in rats is also applicable to humans (Swan *et al.*, 2005; Bustamante-Montes *et al.*, 2013; Curi *et al.*, 2023). In male foetuses, AGD is regulated by testosterone and dihydrotestosterone signalling through the androgen receptor in target tissue (Schwartz *et al.*, 2019). The critical ‘masculinization programming window’ in humans has been identified between gestational weeks 8 and 14 (Welsh *et al.*, 2014). Studies indicate that prenatal exposure to phthalates with anti-androgenic properties during this critical period is associated with a shorter AGD in the offspring (Swan *et al.*, 2005; Bustamante-Montes *et al.*, 2013; Curi *et al.*, 2023). A reduced AGD has been linked to increased risk for cryptorchidism (Eisenberg *et al.*, 2012; Thankamony *et al.*, 2014), hypospadias (Eisenberg *et al.*, 2012; Hsieh *et al.*, 2012; Thankamony *et al.*, 2014), testis cancer (Moreno-Mendoza *et al.*, 2020), poorer semen quality (López-Espín *et al.*, 2018; Priskorn *et al.*, 2019), infertility (Eisenberg *et al.*, 2011), and lower serum testosterone (Eisenberg *et al.*, 2012; Zhou *et al.*, 2016). These reproductive conditions are suggested to be linked with the same underlying cause – testicular dysgenesis syndrome (TDS) (Skakkebaek *et al.*, 2001). The TDS hypothesis suggests that genetic, lifestyle, and environmental factors irreversibly affect the development of the foetal testis. This is supported by the fact that these reproductive conditions are correlated (Skakkebaek *et al.*, 2001, 2016). This study suggests that AGD is established *in utero* and stable through childhood and thereby possibly part of TDS. It may therefore serve as an early, non-invasive, and persistent marker of future male reproductive health. Hence, it is important to follow these children into adulthood to confirm that individual AGD remains stable.

While AGD is a well-established marker in males, particularly in relation to reproductive outcomes, research on its significance in females is comparatively sparse (Schwartz *et al.*, 2019). However, studies report that a longer AGD is associated with higher serum testosterone levels (Mira‐Escolano *et al.*, 2014), a larger number of ovarian follicles (Mendiola *et al.*, 2012), and PCOS (Barrett *et al.*, 2018; Peters *et al.*, 2020; Prieto-Sánchez *et al.*, 2022) in women. In addition, AGD was longer in daughters of women with a PCOS diagnosis compared to daughters of women with no diagnosis (Barrett *et al.*, 2018). A cross-sectional study found that women with shorter AGD were more likely to have received fertility treatment (Wainstock *et al.*, 2017). Additionally, shorter AGD has been linked to endometriosis (Kim *et al.*, 2015; Mendiola *et al.*, 2016; Sánchez‐Ferrer *et al.*, 2019; Peters *et al.*, 2020) and deep infiltrating endometriosis (Mendiola *et al.*, 2016), both of which have been found to impair female fertility (Bulletti *et al.*, 2010). The development of the uterine endometrial gland begins, like AGD, *in utero* and is completed in puberty (Gray *et al.*, 2001), suggesting that endocrine dysfunction can affect fertility *in utero*. Our results suggest that AGD in females remains stable from 3 to 9 years of age, and it can potentially be used as an early predictor of AGD in adulthood.

The long-term longitudinal set-up is a particular strength of the present study. We measured AGD at 6 timepoints from 3 months to 9 years in a large study population. The examinations were mostly carried out by only three technicians, thereby reducing the risk of between-observer variation. The examiners were specially trained and supervised in the AGD and penile width measurements, which increased reliability and reproducibility. Although the measurements were standardized, factors such as the exact position of the child, room temperature, mood of the child, etc. could still impact the measurements. The distance was measured three times, and an average was calculated. The within-observer variation for the different AGD measurements was: AGDas: 4.1%, AGDap: 5.5%, penile width: 11.6%, AGDaf: 6.8%, and AGDac: 6.4% (Priskorn *et al.*, 2018). The between-observer variation for the different AGD measurements was: AGDas: 0.3%, AGDap: 6.8%, penile width: 13.0%, AGDaf: 7.1%, and AGDac: 7.4%.

The fact that the physical landmarks used for the AGD measurements are more distinct in boys (especially the distance between the anus and scrotum, AGDas) may lead to less variation compared to AGD in girls. The larger variation in girls may also be explained by the shorter distance of AGD, making the same absolute measurement error relatively more important. This could explain the weaker correlation coefficients observed between the short AGD measurements (AGDaf) compared to the long AGD measurements (AGDac) in girls and may be a part of the explanation for why the correlations of AGDs were stronger in the older children with longer AGD compared to infants with shorter AGD (as relatively small measurement errors may have a larger impact due to the shorter absolute distance).

AGD is a non-invasive, easily obtained measurement that may be included in routine population studies in children and can provide a baseline for monitoring reproductive health and population trends. Additionally, AGD is used as a biomarker for prenatal exposure to endocrine-disrupting chemicals with anti-androgenic effects. This makes AGD valuable for evaluating exposure to these chemicals in populations. The importance of AGD at the individual level needs further investigations before further diagnostic testing of children with short AGD can be recommended.

In conclusion, in boys, AGD remains stable and tracks from infancy to the age of 9 years, which suggests that it is founded *in utero* and may serve as an early, non-invasive, and sensitive biomarker of male reproductive health. In girls, correlations between AGD increased with increasing age. It is important to follow the children into adulthood to assess if AGD remains stable and to confirm childhood AGD as a biomarker of adult reproductive health.

## Data Availability

The data are available from the corresponding author on reasonable request.
